# Effects of Two Common Polymorphisms rs2910164 in miR-146a and rs11614913 in miR-196a2 on Gastric Cancer Susceptibility

**DOI:** 10.1155/2015/764163

**Published:** 2015-04-23

**Authors:** Qing Ni, Anlai Ji, Junfeng Yin, Xiangjun Wang, Xinnong Liu

**Affiliations:** ^1^Department of General Surgery, First People's Hospital of Yangzhou, Yangzhou, Jiangsu 225001, China; ^2^Department of General Surgery, The Second Medical School of Yangzhou University, Yangzhou, Jiangsu 225001, China

## Abstract

*Background*. Single nucleotide polymorphisms (SNPs) in genes encoding microRNAs may play important role in the development of gastric cancer. It has been reported that common SNPs rs2910164 in miR-146a and rs11614913 in miR-196a2 are associated with susceptibility to gastric cancer. The published results remain inconclusive or even controversial. A meta-analysis was conducted to quantitatively assess potential association between the two common SNPs and gastric cancer risk. *Methods*. A comprehensive literature search was performed in multiple internet-based electronic databases. Data from 12 eligible studies were extracted to estimate pooled odds ratios (ORs) and 95% confidence intervals (95% CI). *Results*. C allele of rs2910164 is associated with reduced gastric cancer risk in heterozygote model and dominant model whereas rs11614913 indicates no significant association. Subgroup analysis demonstrates that C allele of rs2910164 and rs11614913 may decrease susceptibility to diffuse type gastric cancer in dominant model and recessive model, respectively, while rs11614913 increased intestinal type gastric cancer in dominant model. *Conclusion*. SNPs rs2910164 and rs11614913 might have effect on gastric cancer risk in certain genetic models and specific types of cancer. Further well-designed studies should be considered to validate the potential effect.

## 1. Introduction

Gastric cancer is among the leading causes of cancer-related death worldwide. It is estimated that 989,600 new gastric cancer cases were diagnosed in 2008 and caused 738,000 deaths in a single year. Gastric cancer accounts for 8% of total cancer cases and 10% cancer-related death [1]. Despite decreasing incidence of gastric cancer in developed countries, gastric cancer remains a major health problem globally, especially in Eastern Asia, Eastern Europe, and South America, which may be attributed to particular dietary pattern, high prevalence of* Helicobacter pylori* infection, and limited availability to proper food storage [2–4]. The mechanism of gastric carcinogenesis remains elusive. Epidemiological studies have shed light on risk factors of gastric cancer including lifestyle factors, environmental carcinogens, and, importantly,* Helicobacter pylori* infection [5, 6]. However, these risk factors cannot fully explain the development of gastric cancer since only a minority of exposed population finally developed gastric cancer, indicating possible interplay between risk factors and personal background including genetic susceptibility [7].

In recent years, potential association between single nucleotide polymorphisms (SNPs) and risk of gastric cancer were reported [8]. Among the reported SNPs, correlation between SNPs located in genes encoding microRNAs (miRNAs) or their binding sites is of great interest [9, 10]. miRNAs are small noncoding, single-stranded RNA molecules composed of around 22 nucleotides. miRNAs bind to complementary sequences in 3′-untranslated regions of messenger RNAs and negatively regulate their stability or translational efficiency, therefore regulating posttranscriptional activity of genes [11–13]. Aberrant function or expression of miRNAs was reported to play important roles in gastric cancer. Since a single miRNA may have numerous targets, even a slight variation of a miRNA may lead to aberrance of a wide spectrum of gene expression, including many oncogenes and tumor-suppressor genes [7, 14]. SNPs in miRNA may also be involved in gastric cancer susceptibility through altering the expression or function of miRNAs, subsequently leading to aberrant expression of a set of genes [7, 15].

SNPs rs2910164 in miR-146a and rs11614913 in miR-196a2 have been reported as biomarkers of gastric cancer risk [16–27]. However, the results of these studies are controversial and inconclusive. Since the effects of SNPs in miRNAs on gastric cancer susceptibility may be slight, sample size of individual association study could be insufficient to detect minor modifications of gastric cancer risk. In this study, we performed a meta-analysis to systematically estimate the potential association between rs2910164/rs11614913 and susceptibility to gastric cancer with all available evidence.

## 2. Methods

### 2.1. Search Strategy

A systematic literature search was carried out using the combination of the following terms: “miR-146a,” “miR-196a2,” “miR-196a-2,” “rs2910164,” “rs11614913,” “gastric cancer,” “gastric carcinoma,” “gastric adenocarcinoma,” “stomach cancer,” “stomach carcinoma,” and “stomach adenocarcinoma” in multiple databases including PubMed, EMBASE, ISI Web of Knowledge, the Cochrane Library, ScienceDirect, Springer Link, Wiley Online Library, China National Knowledge Infrastructure (CNKI), Wanfang Database, and VIP Info database. Two investigators (Qing Ni and Anlai Ji) independently performed the database search. Publication language, date, and publication form (full-length article or abstract/correspondence) were not restricted. All of the search results were imported into Endnote X6 reference managing software and duplicate records were removed. The reference lists of potentially eligible studies were searched manually. The two investigators crosschecked the search results and reached consensus.

### 2.2. Literature Selection

**Figure 1:**
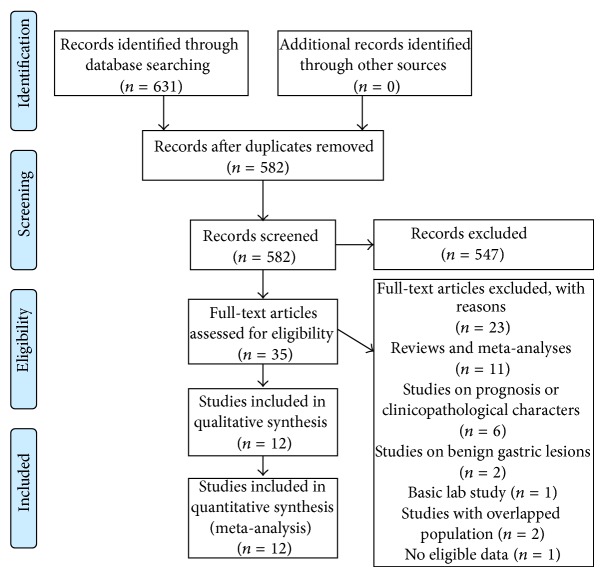
Flow diagram of study search and selection.

We selected eligible studies based on the following criteria: (1) case-control study; (2) investigated associations between rs2910164 and/or rs11614913 and gastric cancer susceptibility; (3) provided sufficient data of allele and genotype frequencies of SNPs or required information could be calculated; (4) if serial studies on the same population were published, only the most recent study was included; (5) proper methodology design. Quality of methodology was evaluated by (1) comparable demographic characteristics between patients and control population; (2) proper diagnosis of gastric cancer; (3) appropriate methods and quality control for genotype determination; (4) Hardy-Weinberg equilibrium that was reached in control group; (5) proper statistical methods that were used. Two independent investigators (Qing Ni and Anlai Ji) performed study selection and reached final consensus. The details of literature search and selection were shown in Figure 1 in standard PRISMA flow diagram style.

### 2.3. Data Extraction

Data for meta-analysis were extracted from eligible studies by two independent investigators (Qing Ni and Anlai Ji). Authors of study, publish year, origin country, ethnicity of studied population, study design (hospital based, HB, or population based, PB), genotyping method, and allele/genotype frequencies were collected. Two investigators crosschecked the results of data abstraction and discussed them to reach mutual agreement by discussion.

### 2.4. Statistical Analysis

Quantitative data synthesis was performed by Review Manager 5.2.11 (Copenhagen: The Nordic Cochrane Centre, The Cochrane Collaboration). Statistical heterogeneity among studies was estimated by *χ* ^2^-based *Q* test. A *P* value less than 0.1 for *Q* test indicated the existence of significant statistical heterogeneity [28]. If no significant heterogeneity was detected, the pooled odds ratios (ORs) with corresponding 95% confidence interval (95% CI) were estimated by the Mantel-Haenszel fixed-effects model [29]. Otherwise, the DerSimonian-Liard random-effects model was used to calculate pooled ORs [30, 31]. The amount of heterogeneity was measured by the *I* ^2^ statistic [32]. *I* ^2^ value less than 25%, between 25% and 50%, greater than 50% indicated low, moderate, and high heterogeneity, respectively. The statistical significance of pooled ORs was determined by *Z* test. A *P* value for *Z* test less than 0.05 was considered statistically significant. Forest plots were provided generated to summarize the results of meta-analysis. The strength of associations between SNPs and the risk of gastric cancer were determined under the following genetic models: (1) allele frequency (C versus G for rs2910164 and C versus T for rs11614913); (2) heterozygous model (GC versus GG for rs2910164 and TC versus TT for rs11614913); (3) homozygous model (CC versus GG for rs2910164 and CC versus TT for rs11614913); (4) dominant model (GC + CC versus GG for rs2910164 and TC + CC versus TT for rs11614913); (5) recessive model (CC versus GG + GC for rs2910164 and CC versus TT + TC for rs11614913).

Sensitivity analysis was conducted by excluding one individual study in turn to observe whether the significance of heterogeneity test and pooled ORs changed. Subgroup analyses were performed by stratified analysis according to Lauren's histology classification of gastric cancer (intestinal or diffuse), cardiac or noncardiac gastric cancer, and lymph node status (N0 or N1) when sufficient data were available.

### 2.5. Publication Bias

Publication bias of the included studies was assessed by funnel plots generated by Review Manager. Begg's test and Egger's test were performed using STATA 11 software. A symmetrical plot suggested no obvious publication bias.

## 3. Results

### 3.1. Characteristics of Included Studies

A total of 582 papers were retrieved after electronic search and duplicate removal. As shown in Figure 1, after initial screening and review of full-text, 12 studies were included in this meta-analysis [16–27]. Characteristics of included studies were presented in Tables 1 and 2. For rs2910164 in miR-146a, 9 studies consisting of 4468 cases and 6844 controls were analyzed [16, 17, 19–22, 24, 25, 27]. For rs11614913, 9 studies involving 3992 cases and 5418 controls were included [16–20, 23, 25–27]. The genotyping methods in these studies include polymerase chain reaction-restriction fragment length polymorphism (PCR-RFLP), polymerase chain reaction with confronting two-pair primers (PCR-CTPP), and TaqMan probe-based genotyping.

### 3.2. Association between rs2910164 in miR-146a and Gastric Cancer Susceptibility

**Figure 2:**
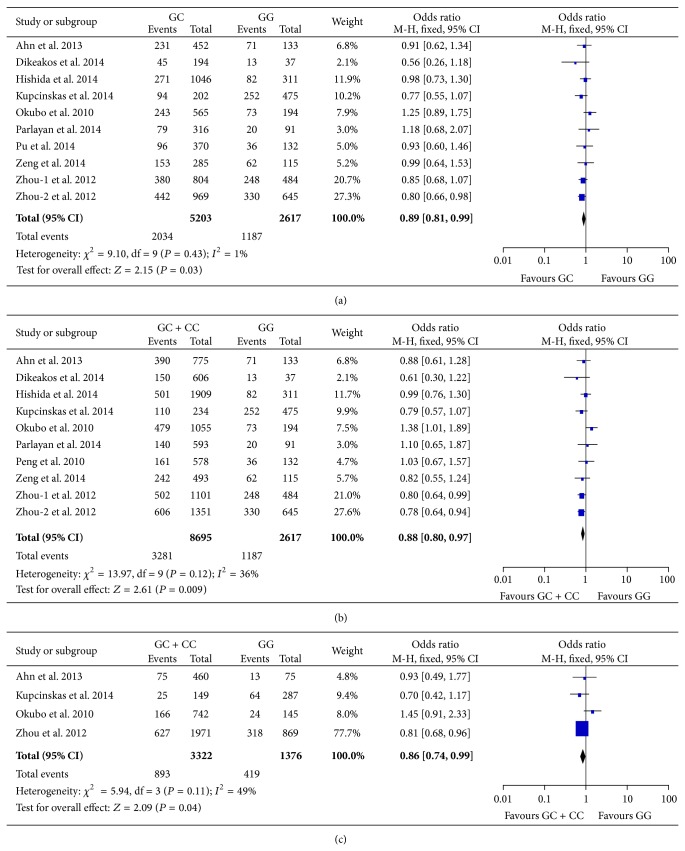
Forest plots of meta-analyses on rs2910164 in miR-146a. (a) Meta-analysis comparing heterozygous GC with GG. (b) Meta-analysis under dominant model (GC + CC versus GG). (c) Subgroup analysis in diffuse type gastric cancer using dominant model (GC + CC versus GG). The blue squares and corresponding horizontal lines indicate odds ratio of individual study. The area of the squares reflects weight of indicated study. The black filled diamond represents pooled odds ratio and 95% confidence interval.

The association between rs2910164 and the risk of gastric cancer were analyzed based on data from 9 studies [16, 17, 19–22, 24, 25, 27]. The report from Zhou et al. [24] is comprised of two independent populations. In this meta-analysis, the two population groups were included separately. Significant heterogeneity was detected in allele frequency model, homozygote model, and recessive model and random-effects model was employed to calculate pooled ORs and 95% CIs in these comparisons. The results of the meta-analyses on rs2910164 were summarized in Table 3. Heterozygous C allele carrier showed decreased risk of gastric cancer compared with GG genotype (OR = 0.89, 95% CI 0.81–0.99, *P* = 0.03, Figure 2(a)). Similarly, in dominant model, GC and CC genotypes were associated with less susceptibility to gastric cancer compared with GG carriers (OR = 0.88, 95% CI 0.80–0.97, *P* = 0.009, Figure 2(b)). No significant association was demonstrated in allele frequency model, homozygote model, and recessive model. Interestingly, in sensitivity analysis, after removal of Okubo et al.'s study, statistical heterogeneity in allele frequency model, homozygote model, and recessive model all became nonsignificant and the pooled ORs showed reduced risk of gastric cancer with statistical significance. Therefore, the study from Okubo et al. may represent an outlier among the included studies. We next performed subgroup analyses stratified by Lauren's histology classification in dominant model and recessive model (Table 5). The results indicated that, in dominant model, GC and CC carriers had reduced risk of diffuse type gastric cancer (OR = 0.86, 95% CI = 0.74–0.99, *P* = 0.04, Figure 2(c)). No significant association was suggested in other models and intestinal type gastric cancer.

### 3.3. Association between rs11614913 in miR-196a2 and Gastric Cancer Susceptibility

**Figure 3:**
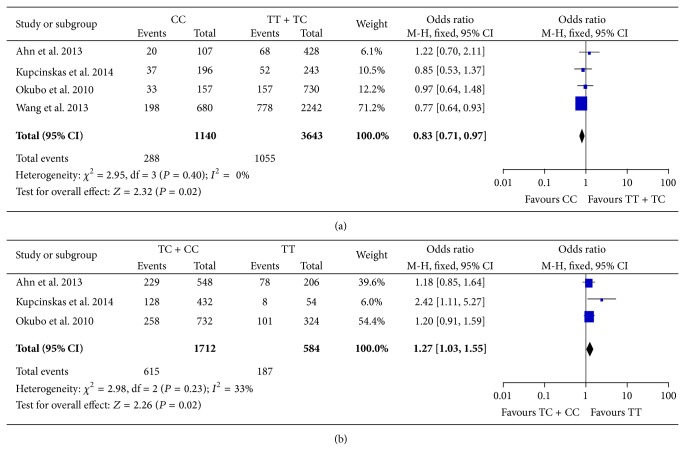
Forest plots of meta-analyses on rs11614913 in miR-196a2. (a) Subgroup analysis in diffuse type of gastric cancer using recessive model (CC versus TT + TC). (b) Subgroup analysis in intestinal type of gastric cancer under dominant model (TC + CC versus TT). The blue squares and corresponding horizontal lines indicate odds ratio of individual study. The area of the squares reflects weight of indicated study. The black filled diamond represents pooled odds ratio and 95% confidence interval.

Potential association between rs11614913 and susceptibility to gastric cancer was evaluated using the data reported in 9 studies [16–20, 23, 25–27]. Wang et al.'s study [23] included two sets of independent cases and controls which were analyzed as separate populations in this meta-analysis. Heterogeneity test in all of the genetic models showed statistical significance and random-effects model was used. The results of the comparisons were listed in Table 4. To our surprise, rs11614913 in miR-196a2 demonstrated no significant association with gastric cancer risk in any genetic model tested. Although exclusion of the studies from Dikeakos et al., Kupcinskas et al., and Wang et al., respectively, diminished statistical heterogeneity in heterozygote model (TC versus CC), pooled ORs remained nonsignificant. In subgroup analyses (Table 5), rs11614913 was not associated with either cardiac or noncardiac lesions. In recessive model, this SNP also presented no association with lymph node metastasis. Interestingly, CC genotype may correlate with a decreased risk of diffuse gastric cancer in recessive model, as suggested by a pooled OR = 0.83 (95% CI 0.71–0.97, *P* = 0.02, Figure 3(a)). TC and CC genotypes may predispose carrier to intestinal type cancer in dominant model (OR = 1.27, 95% CI 1.03–1.55, *P* = 0.02, Figure 3(b)).

### 3.4. Publication Bias

The distribution of studies in funnel plots for analyses of rs2910164 was symmetrical, indicating no evidence for significant publication bias. Begg's test and Egger's test in meta-analyses demonstrating significant outcome also suggested no statistically significant publication bias in these comparisons (GC versus GG: Begg's test *P* = 0.474 and Egger's test *P* = 0.544; GC + CC versus GG: Begg's test *P* = 0.474 and Egger's test *P* = 0.481). However, the funnel plots for rs11614913 showed asymmetrical distribution. Publication bias may exist in studies on potential association between rs11614913 and gastric cancer susceptibility.

## 4. Discussion

Recent research on miRNAs has led to new insight into molecular mechanisms of gastric cancer development [10, 33, 34]. Variations in miRNAs may have profound impact on individual's susceptibility to gastric cancer through regulating a wide spectrum of oncogenes and tumor-suppressor genes. SNPs in miRNA-coding genes and their influence on gastric cancer risk have drawn much attention and related results may help broaden our horizon of gastric cancer. Better understanding of SNPs in miRNAs could improve current management of this detrimental disease by early detection of gastric cancer in high risk populations [35]. Functional SNPs rs2910164 in miR-146a and rs11614913 in miR-196a2 are reported to have association with gastric cancer susceptibility though the results are inconclusive or even controversial [16–27]. In this present study, we conducted a meta-analysis by quantitatively synthesizing available data from 12 published papers to demonstrate potential effects of these two common SNPs on gastric cancer susceptibility.

Located in the stem region opposite to mature miR-146a sequence, rs2910164 G > C polymorphism changed G : U pair to C : U mispair in the stem region of the precursor of miR-146a. C allele of rs2910164 resulted in decreased production of mature miR-146a and subsequently reduced the inhibition of multiple target genes in thyroid cells and hepatocellular carcinoma [36, 37]. In contrast, another two studies reported that C allele of rs2910164 elevated the expression level of miR-146a in breast cancer cells and cervical cancer tissues [38, 39]. The different regulation of this SNP on mature mir-146a may reflect complex gene background between different tissues. The influenced genes by miR-146a include IL-1 receptor-associated kinase 1 (IRAK1), TNF receptor-associated factor 6 (TRAF6), and papillary thyroid carcinoma 1 (PTC1) [36]. Interestingly, IRAK1 and TRAF6 are involved in the regulation of Toll-like receptor (TLR-4) pathway, which has important role in innate immunity against* Helicobacter pylori* [40, 41]. Hishida et al.'s indeed elucidated interaction between miR-146a rs2910164 and TLR4 + 3725 polymorphisms. Their study found that GG genotype of rs2910164 and TLR4 + 3725 C allele increased the risk of severe gastric atrophy in* Helicobacter pylori*-infected Japanese population [21]. miR-146a itself also has important role in cancer cell proliferation [37]. Association between rs2910164 and gastric cancer susceptibility has been reported [17, 20, 22, 24]; however other studies demonstrated no correlation of this SNP with gastric cancer risk [16, 19, 21, 25, 27].

In this meta-analysis, a total of 9 case-control studies were systematically summarized to generate a comprehensive evaluation of the association between rs2910164 in miR-146a and gastric cancer susceptibility. Our result indicated that rs2910164 GC genotype displayed reduced risk of gastric cancer compared with GG carriers. In dominant model, GC and CC genotype also showed decreased susceptibility to gastric cancer with statistical significance. This association was not found in other genetic models. However, the study from Okubo et al. [17] had a significant influence on pooled ORs. In sensitivity analyses, removal of this study not only diminished statistical heterogeneity among included studies but also changed pooled ORs towards significant reduced risk of gastric cancer in allele frequency model (C versus G), homozygote model (CC versus GG), and recessive model (CC versus GC + GG). This study may be the source of heterogeneity with potential bias and could cause a major distortion on the analysis of association between rs2910164 and gastric cancer risk. The possible effect of rs2910164 on gastric cancer susceptibility in allele frequency, homozygote model, and recessive model should not be ruled out. In subgroup analysis, our results demonstrated a significant reduction of diffuse type cancer in dominant model. This result is of considerable importance since diffused type gastric cancer is correlated with poorer prognosis [42]. C allele of rs2910164 may represent a protective factor against diffused type cancer and could serve as a reference in the screening among high risk population.

The other SNP investigated in this study is rs11614913 in miR-196a2. It was initially reported as a prognostic factor of non-small cell lung cancer [43]. The role of rs11614913 in esophageal cancer [44], hepatocellular carcinoma [45], and head and neck cancer [46] was also demonstrated. C allele of rs11614913 increased the expression of mature miR-196a2 in HCC tissues [47] and may cause aberrant expression of downstream genes, including several carcinogenesis-related genes such as homeobox (HOX) family, annexin A1 (ANXA1), and high mobility group AT-hook1 (HMGA1) [48]. Aberrance in HOX family transcription factors plays a significant role in gastric carcinogenesis and cancer stemness [49]. Acting as a mediator of apoptosis and an inhibitor of proliferation, ANXA1 participates in many pathological processes of human disease [50–52]. Deregulation of ANXA1 was found in both precancerous gastric lesions and gastric cancer [53, 54]. Similarly, HMGA1 was also reported to maintain cell proliferation in gastric cancer [55]. Therefore, SNP rs11614913 in miR-196a2 could cause multiple expression change of gastric cancer-related genes and contribute to susceptibility of gastric cancer.

Our meta-analysis systematically summarized data from 9 studies involving 10 study populations and to our surprise, rs11614913 in miR196a2 did not associate with gastric cancer risk in any genetic model tested. Nevertheless, in subgroup analyses, CC genotype of rs11614913 was found to reduce the risk of diffuse type gastric cancer in recessive model compared with TT and TC carriers. Interestingly, TC and CC carriers showed higher risk of intestinal type cancer in dominant model. These findings were not suggested in comparisons in tumor location (cardiac or noncardiac lesion) and lymph node status.

### 4.1. Comparison with Other Meta-Analyses

Before this meta-analysis, several papers from other authors have been published on the effects of rs2910164 and rs11614913 on cancer risk [56–68]. However, most of these studies did not distinguish type of cancers and investigated overall effect of the SNPs on all types of cancer [56–59, 62]. Some papers narrowed the aim of study to digestive cancers or gastrointestinal cancers but still included several cancers from different tissues [60, 61, 64–66]. A major concern is that different cancers from different tissue origins have distinct mechanism of pathogenesis. The clinical heterogeneity brought by this inherent difference could distort the result of meta-analysis. Only one study from Hua et al. summarized potential effect of these two common SNPs on gastric cancer by meta-analysis [7]. Their study found no association between rs2910164 and rs11614913 and gastric cancer susceptibility. Several additional studies have been reported after they published their paper, which is added to this updated meta-analysis. Therefore, this present study included all available evidence up to date and provided most comprehensive analysis regarding the effect of these two common SNPs on gastric cancer risk. Additionally, we also performed subgroup analyses to explore potential association between SNPs and cancer histological types, tumor locations, and lymph node status. Our results may expand our knowledge on rs2910164 and rs11614913 and their role in altering the risk of gastric cancer.

### 4.2. Limitations

Of note, this meta-analysis has its limitations and the results should be interpreted with caution. First, although we carried out the comparisons in similar backgrounds, significant heterogeneity still exists, especially in the analyses of rs11614913. Okubo et al.'s study [17] brought statistical heterogeneity with significance in the comparisons of rs2910164. The heterogeneity may distort the results of this meta-analysis and potential association between rs2910164 and susceptibility to gastric cancer should not be ruled out in genetic models that did not derive statistical significance. Second, due to limited number of studies, the subgroup analyses should be interpreted cautiously even if they indicated positive results. The role of these SNPs in different histological types should be further explored. Third, publication bias existed in studies on rs11614913, which implies the true effect of rs11614913 may not be fully discovered or reported.

In summary, despite the limitations, this meta-analysis suggests that rs2910164 in miR-146a and rs11614913 in miR-196a2 might be associated with reduced gastric cancer risk in certain genetic models and cancer histological types. More future studies with good methodology design are warranted.

## Figures and Tables

**Table 1:** Characteristics of included studies on rs2910164.

Author	Year	Country	Ethnicity	Study design	SNP	Genotyping methods	HWE	Case genotype			Control genotype		
								GG	GC	CC	GG	GC	CC
Ahn et al. [20]	2013	South Korea	Asian	HB	rs2910164	PCR-RFLP	0.362	71	231	159	62	221	164
Dikeakos et al. [19]	2014	Greece	Caucasian	HB	rs2910164	PCR-RFLP	0.289	13	45	105	24	149	307
Hishida et al. [21]	2011	Japan	Asian	HB	rs2910164	PCR-CTPP	0.738	82	271	230	229	775	633
Kupcinskas et al. [25]	2014	Germany/ Lithuania/ Latvia	Caucasian	HB	rs2910164	TaqMan	0.531	252	94	16	223	108	16
Okubo et al. [17]	2010	Japan	Asian	HB	rs2910164	PCR-RFLP	0.278	73	243	236	121	322	254
Parlayan et al. [16]	2014	Japan	Asian	HB	rs2910164	TaqMan	0.640	20	79	61	71	237	216
Pu et al. [27]	2014	China	Asian	HB	rs2910164	PCR-RFLP	0.080	36	96	65	96	274	143
Zeng et al. [22]	2010	China	Asian	HB	rs2910164	PCR-RFLP	0.122	62	153	89	53	132	119
Zhou et al. [24]	2012	China	Asian	HB	rs2910164	TaqMan	0.544	248	380	122	236	424	175
							0.929	330	442	164	315	527	218

SNP, single nucleotide polymorphism; HB, hospital based; PCR-RFLP, polymerase chain reaction-restriction fragment length polymorphism; PCR-CTPP, polymerase chain reaction with confronting two-pair primers; HWE, *P* value for Hardy-Weinberg equilibrium test.

**Table 2:** Characteristics of included studies on rs11614913.

Author	Year	Country	Ethnicity	Study design	SNP	Genotyping methods	HWE	Case genotype			Control genotype		
								TT	TC	CC	TT	TC	CC
Ahn et al. [20]	2013	South Korea	Asian	HB	rs11614913	PCR-RFLP	0.322	119	242	100	128	232	87
Dikeakos et al. [19]	2014	Greece	Caucasian	HB	rs11614913	PCR-RFLP	0.850	15	46	102	172	229	79
Kupcinskas et al. [25]	2014	Germany/ Lithuania/ Latvia	Caucasian	HB	rs11614913	TaqMan	0.161	35	184	144	46	145	159
Okubo et al. [17]	2010	Japan	Asian	HB	rs11614913	PCR-RFLP	0.510	166	281	105	223	350	124
Parlayan et al. [16]	2014	Japan	Asian	HB	rs11614913	TaqMan	0.410	44	72	44	146	270	108
Peng et al. [18]	2010	China	Asian	HB	rs11614913	PCR-RFLP	0.936	43	94	76	50	107	56
Pu et al. [27]	2014	China	Asian	HB	rs11614913	PCR-RFLP	<0.01	25	95	39	86	324	101
Wang et al. [66]	2013	China	Asian	HB	rs11614913	TaqMan	0.898	226	371	152	232	448	220
							0.058	293	480	167	292	492	262
Yang et al. [26]	2013	China	Asian	PB	rs11614913	TaqMan	0.100	21	109	102	42	136	72

SNP, single nucleotide polymorphism; HB, hospital based; PB, population based; PCR-RFLP, polymerase chain reaction-restriction fragment length polymorphism; HWE, *P* value for Hardy-Weinberg equilibrium test.

**Table 3:** Summary of pooled ORs in meta-analyses of rs2910164.

Genetic model	Pooled OR [95% CI]	*P*	*P*_hetero_
C versus G	0.94 [0.85–1.04]	0.21	0.003
GC versus GG	0.89 [0.81–0.99]	0.03	0.43
CC versus GG	0.89 [0.72–1.08]	0.23	0.009
GC + CC versus GG	0.88 [0.80–0.97]	0.009	0.12
CC versus GC + GG	0.94 [0.81–1.08]	0.38	0.008

OR, odds ratio; 95% CI, 95% confidence interval; *P*_hetero_, *P* value for heterogeneity test.

**Table 4:** Summary of pooled ORs in meta-analyses of rs11614913.

Genetic model	Pooled OR [95% CI]	*P*	*P*_hetero_
C versus T	1.25 [0.97–1.60]	0.09	<0.00001
TC versus TT	1.09 [0.94–1.28]	0.25	0.06
CC versus TT	1.52 [0.96–2.39]	0.07	<0.00001
TC + CC versus TT	1.26 [0.98–1.63]	0.07	<0.00001
CC versus TC + TT	1.36 [0.90–2.05]	0.14	<0.00001

OR, odds ratio; 95% CI, 95% confidence interval; *P*_hetero_, *P* value for heterogeneity test.

**Table 5:** Summary of pooled ORs in subgroup analyses.

SNP	Number of studies	Subgroup	Genetic model	Pooled OR [95% CI]	*P*	*P*_hetero_
rs2910164	4	Intestinal type	GC + CC versus GG	0.95 [0.72–1.25]	0.7	0.04
rs2910165	4	Diffuse type	GC + CC versus GG	0.86 [0.74–0.99]	0.04	0.11
rs2910164	3	Intestinal type	CC versus GG + GC	0.91 [0.76–1.11]	0.36	0.33
rs2910165	3	Diffuse type	CC versus GG + GC	0.88 [0.68–1.14]	0.33	0.26
rs11614913	3	Cardiac lesion	CC versus TT + TC	0.91 [0.51–1.64]	0.76	0.04
rs11614913	3	Noncardiac lesion	CC versus TT + TC	1.10 [0.63–1.89]	0.74	0.0002
rs11614913	3	Lymph node negative	CC versus TT + TC	0.89 [0.74–1.07]	0.22	0.15
rs11614913	3	Lymph node positive	CC versus TT + TC	1.54 [0.54–4.35]	0.42	<0.00001
rs11614913	3	Intestinal type	TC + CC versus TT	1.27 [1.03–1.55]	0.02	0.23
rs11614913	3	Diffuse type	TC + CC versus TT	1.01 [0.78–1.32]	0.92	0.55
rs11614913	4	Intestinal type	CC versus TT + TC	0.91 [0.64–1.28]	0.58	0.003
rs11614913	4	Diffuse type	CC versus TT + TC	0.83 [0.71–0.97]	0.02	0.4

OR, odds ratio; 95% CI, 95% confidence interval; *P*_hetero_, *P* value for heterogeneity test.
